# Herd and cow characteristics affecting the odds of veterinary treatment for disease – a multilevel analysis

**DOI:** 10.1186/1751-0147-51-34

**Published:** 2009-08-22

**Authors:** Marie Jansson Mörk, Ulf Emanuelson, Ann Lindberg, Ivar Vågsholm, Agneta Egenvall

**Affiliations:** 1 Department of Clinical Sciences, Swedish University of Agricultural Sciences, PO Box 7019, SE-750 07 Uppsala, Sweden; 2 National Veterinary Institute, SE-751 89, Uppsala, Sweden

## Abstract

**Background:**

Research has indicated that a number of different factors affect whether an animal receives treatment or not when diseased. The aim of this paper was to evaluate if herd or individual animal characteristics influence whether cattle receives veterinary treatment for disease, and thereby also introduce misclassification in the disease recording system.

**Methods:**

The data consisted mainly of disease events reported by farmers during 2004. We modelled odds of receiving veterinary treatment when diseased, using two-level logistic regression models for cows and young animals (calves and heifers), respectively. Model parameters were estimated using three procedures, because these procedures have been shown, under some conditions, to produce biased estimates for multi-level models with binary outcomes.

**Results:**

Cows located in herds mainly consisting of Swedish Holstein cows had higher odds for veterinary treatment than cows in herds mainly consisting of Swedish Red cows. Cows with a disease event early in lactation had higher odds for treatment than when the event occurred later in lactation. There were also higher odds for veterinary treatment of events for cows in January and April than in July and October. The odds for veterinary treatment of events in young animals were higher if the farmer appeared to be good at keeping records. Having a disease event at the same date as another animal increased the odds for veterinary treatment for all events in young animals, and for lameness, metabolic, udder and other disorders, but not for peripartum disorders, in cows. There were also differences in the odds for veterinary treatment between disease complexes, both for cows and young animals.

The random effect of herd was significant in both models and accounted for 40–44% of the variation in the cow model and 30–46% in the young animal model.

**Conclusion:**

We conclude that cow and herd characteristics influence the odds for veterinary treatment and that this might bias the results from studies using data from the cattle disease database based on veterinary practice records.

## Background

Previous research has indicated that a number of different factors may influence the farmers' treatment decisions for diseased dairy cattle, and whether a veterinarian is contacted or not. Nyman et al. [1] noted that the threshold for contacting the veterinarian differed between dairy farmers in Sweden. Moreover, Vaarst et al. [2] found that the decision about veterinary treatment in Danish dairy cows depended not only on the disease event's severity, but also on the age of the cow, lactational stage, milk yield and/or the temperament of the cow, and that the farmers weighted these factors differently. The economic value of an animal is also likely to affect the decision about veterinary treatment. The individual dairy cows' retention pay-off values differ depending on, for example, their parity, stage of lactation and milk yield [3]. Another example is the report by Ortman and Svensson [4] where they found a high proportion of treatments in young animals initiated by the farmers themselves. How farmers' decisions about treatments can influence data quality is exemplified by Mulder and colleagues [5] who compared cows with complete data records (defined as "not having missing data for postpartum evaluation, pregnancy diagnosis and body condition score...") versus missing data records and found a lower reproductive performance in cows with complete data records. One possible explanation was that problem-cows were identified early, and treated more intensively.

These findings indicate that cow, herd and/or farmer characteristics may affect whether an animal receives treatment or not when diseased. As a consequence of this, misclassification of disease events in animal disease recording systems based on veterinary treatments could be differential, i.e. occur with different magnitudes and with different directions. Note that the source of misclassification in this report is data loss – because animals are classified as healthy when there are no records saying they are diseased. Secondary databases with disease information have been used for research in several scientific areas, such as epidemiology, genetics and animal health economics [6-8]. The advantage of such secondary databases is the large amount of data available at a low cost. However, a disadvantage is that the researcher does not have control over the data collection and consequently not of the data quality either. To ensure the data quality, a secondary database needs to be validated [9-11].

The dairy disease database (DDD) at the Swedish Dairy Association is based primarily on clinical disease events reported by veterinarians and is used for sire evaluation, extension services, annual statistics and research. The DDD has been evaluated concerning completeness with respect to all the disease events observed by farmers [12] and also in respect of disease events resulting in veterinary treatment (Mörk et al., unpublished). It was found that only 54% of the disease events detected by farmers were treated by veterinarians. Consequently, the incidence rates for different disease complexes, based on the reported events, were significantly lower compared to the incidence rates based on farmer observations. It would be of interest to characterize this loss of data by examining if any animal and herd factors influenced whether a diseased animal received veterinary treatment or not.

Hence, the objective of this study was to evaluate if herd or individual animal characteristics influence whether a cow or young animal receives veterinary treatment for disease, and thereby also introduce differential misclassification in the disease recording system.

## Materials and methods

### Study population and design

The study population and data collection have been described previously [12]. In brief, a baseline study of disease incidence in dairy farms, based on farmers' records, was performed during January, April, July and October in 2004. Four-hundred herds were randomly selected from all the herds enrolled in the Swedish Official Milk Recording Scheme, which included about 86% of the Swedish dairy cows in 2004, and 177 participated.

The farmers were asked to record disease events, defined as an observed deviation in health. The farmer could either choose to wait, treat the animals him/herself, contact a veterinarian for diagnosis and treatment, or slaughter the animal. The data reported for each disease event were as follows: the animal's identity and sex, the date when the disease was observed, the diagnosis, whether or not a veterinarian was consulted, the farmer's description of the event (e.g. symptoms) and the treatment given. The farmers could use the following diagnoses: acetonemia/inappetence, abomasal displacement, calving problems, clinical mastitis, clinical puerperal paresis, coughing, diarrhoea, lameness (of a hoof), lameness (of a limb), retained placenta and other diseases. During data editing the diagnosis "other disease" was categorised into gastro-intestinal disorders, laminitis, paresis (not puerperal), peripartum disorders (retained placenta and puerperal paresis not included), ringworm/lice, traumatic reticuloperitonitis, udder disorders and other disorders based on the descriptions provided by the farmer. The farmer did not have to report the animals' identities for events where groups of animals were affected.

When comparing the disease events reported by the farmers with those reported by veterinarians in the DDD, it could be observed that some events resulting in veterinary treatment had not been reported to us by the farmers [12]. Such events were also included in the analyses in this study.

### Data from the Swedish Official Milk Recording Scheme

Information about the herds studied was obtained from the Milk Recording Scheme at the Swedish Dairy Association in November 2005. The data consisted of herd characteristics, such as the housing system and the herd size, as well as individual cow parameters, such as parity, calving dates, milk yield, fertility treatments and disease events.

### Data editing

The herds were categorised as Swedish Red (SR) herds or Swedish Holstein (SH) herds if at least 80% of the animals were pure-bred SR or SH, respectively, and as mixed/other breeds otherwise. Further herd-level characteristics were: the average milk yield in 2003 (calculated as the total daily milk yield in the herd/total number of cow-days for lactating cows), the average parity, the proportion of older cows (above the 2^nd ^and 3^rd ^lactation, respectively), the average somatic-cell count (SCC) in test milk, the average udder-disease score (UDS) and the proportion of cows with a high UDS, indicative of sub-clinical mastitis. The UDS is used to measure the probability of a cow having mastitis and is based upon a series of three test day SCC results at monthly intervals for individual cow's SCC [13]. The variables were checked for implausible values, but none were found.

The data on animals with disease events reported by the farmers were merged with data from the milk recording scheme and the DDD and categorised into young animals (prior to the first calving for heifers) and cows, respectively. All the bulls with a reported disease event were below 2 months of age. The following cow characteristics were available: the breed, the parity, the milk yield on the test day prior to the disease event, the average SCC and average UDS, for the past 305-day period and 90-day period respectively, the days in milk at the disease event, the state of pregnancy at the time of the disease event (yes/no) and the number of inseminations prior to previous and current pregnancy. Information was also available as to whether the cow was culled or not after the disease event (not culled, culled within lactation, culled after the current lactation) and the reported reason for culling. For young animals, the age at the time of the disease and the breed were available in the data from the Milk Recording Scheme.

In the current study, for cows, the disorders were categorised into the following disease complexes: lameness, metabolic, peripartum disorders (puerperal paresis not included), udder disorders and other disorders; and for young animals: coughing, diarrhoea, lameness and other disorders. Only four young animals had udder disorders, and these were categorised as other disorders.

In a previous study we found that the study farmers reported only 88% of the disease events reported to the DDD (by veterinarians) to us [12]. Based on that finding, the farmers with an apparently good record-keeping ability were identified. Farmers qualifying as good record keepers had accomplished one of the following: i) they had reported all the events registered in the DDD (by veterinarians), or ii) they had failed to report one event registered in the DDD, but the number of successfully reported events was > 1, or iii) they had failed to report ≥ 2 events registered in the DDD, but the proportion of successfully reported events was > 0.75. The reasoning behind the different definition for farmers with one and more than one event missing was that one event was thought to be easily forgotten without necessarily indicating a waning interest in the study.

### Missing data

Fifteen animals were dropped because data on them were missing. Moreover, for 34 cows with incomplete calving data, an approximate calving date was calculated for estimation of the milk yield in the latest 305 day-period. This was accomplished by subtracting the study population's median calving interval (384 days) from the following calving date.

### Statistical analysis

Two-level regression models were fitted with a logit-link. The dependent variable was whether the diagnostic event had resulted in veterinary treatment (yes = 1/no = 0), as reported by the farmers (or the DDD). Veterinary treated refers to all events where the farmer contacted a veterinarian, even if no medical treatment was delivered. The probability (*p*^veterinary treatment^) of veterinary treatment for an animal with a diagnostic event depended on explanatory variables (*x*_1_,..., *x*_*n*_) and on the random effect of herd (*u*_herd_). The random effect was assumed to be independently and normally distributed with a standard deviation, *σ*_*u*_. The model for animal *i *was expressed (using logit *(p) *= log *(p/(1 - p))) *as



where *β*_0*j *_= *β*_0 _+ *u*_0(herd j)_

The data on cows and young animals were analysed separately in two models. Only events where the animal's identity number was reported were used in the analysis (i.e. no events reported for groups. The group reported events were: eight and nine events of herd-outbreaks of cough and diarrhoea, respectively). Further, 326 cows had more than one diagnostic event, either at the same date or at different dates. Only one diagnostic event per cow was included in the analysis to avoid the effects of clustering on the individual animal level. These events were selected by giving the events a random number and including the one with the lowest number. Moreover, we included in our models only disease events from herds with at least four disease events in dairy cows or young animals, respectively.

The continuous variables, except the herd's average milk yield, were not linearly related to the outcome (based on logit-transformed smoothed scatterplots) and therefore categorised using the 25^th^, 50^th^, and 75^th ^percentiles. The association between the outcome and each potential fixed explanatory variable was tested in a univariable analysis including herd as a random effect. By including a dispersion parameter in the empty two-level models, we estimated the extra-binomial variation to be 0.86 for cows and 0.94 for young animals. Since it was reasonably close to 1, the dispersion parameter was not considered in further analyses. The final models were, however, re-fitted with the dispersion parameter included, resulting in no changes in the estimates in the cow model and only small (less than 0.1) changes in the estimates in the young animal model and only results from the models without the dispersion parameter is presented. Correlations between the explanatory variables considered for further analysis were investigated using Spearman correlation coefficients, with the intention of dropping one of the variables if the correlation was ≥ 0.7 or ≤ -0.7. In the analyses for cows, the herd's proportion of cows above the third lactation and the herd's average parity had a correlation coefficient of 0.8 and the herd's average parity was therefore excluded in the multivariable analysis. In the analyses for young animals, no variables were dropped.

All the explanatory variables with a p-value < 0.2 (in the likelihood ratio test) in the univariable analyses and no missing observations were included in the multivariable analysis. The model was reduced manually by backward elimination. A variable with a p-value ≤ 0.05 (in the likelihood ratio test) was considered statistically significant and kept in the final model. All the variables excluded were then re-entered, one at a time, and kept if their p-value was ≤ 0.05. All the two-way interactions were then tested for inclusion one by one. A variable was considered to be a confounder, and therefore retained in the model regardless of significance tests, if deleting it from the model resulted in the change of another parameter estimate by more than 20% [14]. The variance partition coefficient (VPC) was estimated by (σ^2^_herd-level_/σ^2^_herd-level _+ σ^2^_event-level_), where we assumed that the level-one (event) variance was π^2^/3 (where π = 3.1416) on the logit scale [15].

Data editing, descriptive statistics and model building (log likelihood estimation (LL) using the xtmelogit command) were performed in Stata^®^version 10 (Stata Corporation, College Station, TX, USA). The final models were also estimated using the second-order penalized quasi-likelihood (PQL) and the restricted iterative generalised square algorithm and the Markov-chain Monte Carlo (MCMC) procedures in MLwiN (version 2.1, Institute of Education, University of London, UK). The evaluation of extra binomial variation was performed using the PQL estimation. The MCMC model was fitted using the Metropolis-Hastings algorithm with diffuse priors, a burn-in length of 500 iterations and a monitoring period of 90,000 iterations. The model fit was evaluated by plotting the standardized residuals against the fixed part prediction and normal scores, respectively, at the second level (herd) for the PQL estimation. For the cow model and the young animal model, the points in the plot of standardized residuals against the fixed part prediction showed an equal-width band and the plot of standardized residuals against normal scores showed a, roughly, straight line. For the cow model, two possible outliers (standardized residual below -3) were detected but the model did not change much when those observations were deleted.

## Results

### Description of datasets

In the original data for cows there were 2,112 diagnostic events in 171 herds. From these data, 338 events were deleted because of multiple events per cow. Moreover, 67 events in 31 herds were deleted because the herds had less than four events. The original young animal data contained 362 diagnostic events in 96 herds, of which 13 diagnostic events were deleted because of multiple events in one animal. Another 106 events and 68 herds were removed because the herds had less than four events. The resulting datasets consisted of 1,707 diagnostic events (in 140 herds) in cows and 243 diagnostic events (in 28 herds) in young animals.

For cows the average number of events per herd was 12.2 (with the median being 10, the range 4–88). Of all the events, the proportion that resulted in veterinary treatment per herd ranged between 0% and 100%, with the 10^th^, 50^th ^and 90^th ^percentiles being 21%, 75% and 100%. For young animals the average number of events per herd was 8.7 (with the median being 6, the range 4–37). The percentage of events resulting in veterinary treatment per herd ranged between 0% and 100%, with the 10^th^, 50^th ^and 90^th ^percentiles being 0%, 18% and 100%, respectively

### Logistic regression analysis

#### Cows

For cows, the categorical variables included in the multivariable analysis are presented in Table 1. Variables with a p-value > 0.2 in the initial analysis, and thus not included were: good record-keeping ability, herd average SCC, herd size, parity, private or state-employed veterinary district, and the proportion of cows older than the second lactation.

**Table 1:** Distribution of disease events according to categorical variables potentially associated with the odds of veterinary treatment for young animal and cows, respectively.

Variables	Categories	No. of herds	No. of events	Veterinary treatment	
				No.	%
**Young animals:**					
*Herd level*					
Average SCC in test-milk	< 170	11	96	20	21
	170–196	4	42	7	17
	196.1–236	9	69	27	39
	> 236	4	36	14	39
Average udder disease score ^a^	< 2.4	7	74	22	30
	2.4–2.6	7	47	12	26
	2.61–2.7	4	38	7	18
	> 2.7	10	84	27	32
Main breed	Swedish Red	10	74	11	15
	Swedish Holstein	18	169	57	34
Good record-keeping ability	Yes	24	217	53	24
	No	4	26	15	58
Participation	Only January	2	11	5	45
	All months	26	232	63	27
Veterinary district	Private	11	84	16	19
	State-employed	17	159	52	33
*Disease event level*					
Age	< 2 months	24	149	26	17
	2–15 months	17	54	14	26
	> 15 months	20	40	28	70
Study month	January	25	116	26	22
	April	16	37	14	38
	July	18	45	11	24
	October	19	45	17	38
Breed	Swedish Red	15	65	6	9
	SH	23	167	57	34
	Mixed/Other	6	11	5	45
Disease	Cough	12	52	17	33
	Gastro-intestinal disorders	22	96	7	7
	Lameness disorders	14	28	19	68
	Other disorders	17	67	25	37
**Cows:**					
*Herd level*					
Main Breed	Swedish Red	60	617	387	63
	Swedish Holstein	65	913	689	75
	Mixed/Other	15	177	123	69
Housing type	Tied	107	1,066	721	68
	Warm loose	27	175	382	218
	Cold loose	6	466	96	21
Proportion of cows above third lactation	< 0.14	35	429	302	70
	0.14–0.179	26	416	267	64
	0.18–0.24	39	430	313	73
	> 0.24	40	432	317	73
*Disease event level*					
Another animal with an event at the same date	Yes	118	726	594	82
	No	140	981	605	62
Breed	Swedish Red	105	633	404	64
	Swedish Holstein	113	996	742	74
	Other	35	78	53	68
Culled	Not culled	129	690	510	74
	≤ 305 days after event	136	736	505	69
	> 305 days after event	115	281	184	65
Days in milk	< 7	125	394	318	81
	7–69	126	450	327	73
	70–168	125	435	292	67
	> 168	119	428	262	61
Disease complex	Lameness disorders	90	316	166	53
	Metabolic disorders	112	260	200	77
	Other disorders	78	144	111	77
	Peripartum disorders	77	124	96	77
	Udder disorders	138	863	626	73
Study month	January	128	519	317	61
	April	120	353	227	64
	July	111	436	349	80
	October	116	399	306	77
Pregnant	Yes	91	237	161	68
	No	140	1,470	1,038	71
Reason for culling	Not culled	129	691	510	74
	Milk	56	102	60	59
	Udder	121	405	281	69
	Other	128	509	348	68

^a ^The udder disease score is used to measure the probability of a cow having mastitis and is based upon a series of three monthly test day SCC results for the individual-cow [13].

The only continuous variable included in the multivariable analysis was the herd's average milk yield. The 10^th^, 50^th ^and 90^th ^percentiles for the herd's average milk yield (kg of energy-corrected milk) were 6,564, 7,903 and 9,344 for herds for which events resulting in veterinary treatment had been reported, and 6,313, 7,751 and 9,303 for herds for which events resulting in veterinary treatment had not been reported. The average milk yields per cow in the latest 305-day and 90-day periods had a p-value < 0.2 in the univariable analysis, but were not included in the multivariable analysis because of missing observations. Instead, cow average milk yield in the latest 305-day and 90-day periods were tested in a model containing the explanatory variables that remained in the final model. They were, however, not statistically significant.

One herd-level variable and four event-level variables were retained in the final model for cows. Moreover, the final model included an interaction between the disease complex and another animal with an event at the same date. The estimates and standard errors based on the LL, PQL and MCMC procedures were similar (Table 2).

**Table 2:** Explanatory variables significantly associated with veterinary treatment (yes = 1/no = 0), given a disease event (two-level logistic model) for cows using different estimating algorithms.

		LL ^a^		PQL ^b^		MCMC ^c^	
Variables	Categories	Estimate	SE	Estimate	SE	Estimate	SE
*Fixed part:*							
Intercept		1.37	0.32	1.32	0.31	1.41	0.33
Another animal with an event at the same date	Yes	2.18	0.26	2.18	0.27	2.22	0.26
	No	0		0		0	
Breed^e^	Swedish Holstein	0		0		0	
	Swedish Red	-0.82	0.33	-0.79	0.33	-0.85	0.35
	Other/mixed	-0.86	0.52	-0.83	0.51	-0.90	0.54
Days in milk	< 7	0		0		0	
	7–69	-0.52	0.23	-0.53	0.23	-0.54	0.23
	70–168	-0.93	0.24	-0.93	0.24	-0.95	0.24
	> 168	-1.12	0.24	-1.13	0.24	-1.15	0.24
Disease complex	Udder	0		0		0	
	Metabolic	0.09	0.25	0.09	0.25	0.10	0.26
	Lameness	-0.91	0.26	-0.91	0.26	-0.92	0.26
	Reproductive	0.37	0.39	0.36	0.39	0.39	0.39
	Other	0.16	0.34	0.16	0.33	0.17	0.34
Study month	January	0		0		0	
	April	0.31	0.19	0.31	0.19	0.31	0.20
	July	1.31	0.20	1.31	0.20	1.33	0.21
	October	1.20	0.20	1.20	0.20	1.23	0.21
Disease complex X Another animal with an event at the same date	Metabolic X Yes	-0.88	0.51	-0.88	0.51	-0.88	0.51
	Lameness X Yes	-1.56	0.39	-1.55	0.39	-1.59	0.39
	Reproductive X Yes	-2.13	0.62	-2.12	0.62	-2.15	0.63
	Other X Yes	-0.04	0.66	-0.03	0.67	0.02	0.67
*Random part:*							
Herd		2.33	0.48	2.26	0.38	2.61	0.54

^a ^Log likelihood.

^b ^Second-order penalized quasi-likelihood (PQL) estimates with restricted iterative generalised square algorithm.

^c ^Markov-chain Monte Carlo (MCMC) estimates.

^d ^Herd-level variable.

The odds ratios for veterinary treatment for the LL estimation are presented in Table 3. The interaction term is presented as a comparison within the disease complex in Table 3. The baseline for the interaction term was udder disorders combined with no other event at the same day. When no other animal in the herd had an event at the same date, lameness disorders had a statistically significantly lower odds for veterinary treatment than the other disease complexes (OR 0.37; 95% confidence interval (CI) 0.19, 0.70 compared to metabolic disorders; OR 0.35; 95% CI 0.16, 0.74 compared to other disorders; OR 0.28; 95% CI 0.12, 0.67 compared to peripartum disorders and OR 0.40; 95% CI 0.24, 0.67 compared to udder disorders). When another animal had an event at the same date, lameness disorders had a significantly lower odds for veterinary treatment than metabolic disorders (OR 0.19; 95% CI 0.08, 0.45), other disorders (OR 0.08; 95% CI 0.03, 0.28) and udder disorders (OR 0.09; 95% CI 0.05, 0.16), and peripartum disorders had a significantly lower OR than udder disorders (OR 0.17; 95% CI 0.06, 0.48) and other disorders (OR 0.15; 95% CI 0.04, 0.61).

**Table 3:** Odds ratios (ORs) with 95% confidence intervals (CIs) for the explanatory variables significantly associated with veterinary treatment (yes = 1/no = 0), given a disease event (two-level logistic model) for cows estimated using log likelihood estimation.

Variables	Categories	OR	95% CI	
*Fixed part:*				
Breed ^a^	Swedish Holstein	BL^b^		
	Swedish Red	0.4	0.2	0.8
	Mixed/other	0.4	0.2	1.2
Days in milk	< 7	BL		
	7–70	0.6	0.4	0.9
	70–168	0.4	0.3	0.6
	> 168	0.3	0.2	0.5
Study month	January	BL		
	April	1.4	0.9	2.0
	July	3.7	2.5	5.5
	October	3.3	2.2	4.9
Disease complex X. Another animal with an event at the same date. ^c^	Udder X No	BL		
	Udder X Yes	8.8	5.3	14.8
	Metabolic X No	BL		
	Metabolic X Yes	3.7	1.5	8.7
	Lameness X No	BL		
	Lameness X Yes	1.9	1.0	3.4
	Reproductive X No	BL		
	Reproductive X Yes	1.1	0.4	3.2
	Other X No	BL		
	Other X Yes	8.5	2.6	27.9

^a ^Herd-level variable.

^b ^Baseline.

^c ^OR only comparable within disease complex.

Herd as a random factor was significant and accounted for 41% of the modelled variation in the LL estimation and 41% and 44% in the PQL and MCMC estimations, respectively.

#### Young animals

The herd's average milk yield, the herd's average UDS, herd size, housing type and the proportion of cows older than the second and third lactation, respectively, were tested in the initial analysis for young animals, but had p-values > 0.2. The categorical candidate variables included in the multivariable analyses are presented in Table 1.

The different estimation procedures showed different results for the young animals' analysis, with lower estimates for most variables in the LL procedure (Table 4). The ORs for the LL procedure are presented in Table 5. Study month was identified as a confounder and was included in the final model although non-significant (p = 0.07, data not shown).

**Table 4:** Explanatory variables^a ^significantly associated with veterinary treatment (yes = 1/no = 0), given a disease event (two-level logistic model) for young animals using different estimating algorithms.

		LL^b^		PQL ^c^		MCMC ^d^	
Variables	Categories	Estimate	SE	Estimate	SE	Estimate	SE
*Fixed part:*							
Intercept		-4.51	0.76	-4.71	0.82	-5.01	0.84
Age	< 2	0		0		0	
	2 to 15	0.62	0.58	0.68	0.61	0.73	0.62
	> 15	2.02	0.71	2.20	0.70	2.36	0.76
Another animal with an event at the same date	Yes	1.30	0.48	1.38	0.50	1.47	0.52
	No	0		0		0	
Disease complex	Lameness disorders	2.43	0.81	2.40	0.85	2.55	0.88
	Other disorders	1.80	0.63	1.84	0.67	1.95	0.68
	Cough	1.48	0.72	1.49	0.77	1.60	0.79
	Gastro-intestinal disorders	0		0		0	
Good record-keeping ability	Yes	0		0		0	
	No	2.34	0.95	2.45	1.08	2.64	1.18
*Random part:*							
Herd		1.39	0.89	2.02	0.96	2.81	1.86

^a ^Study month was identified as a confounder and was therefore also included in the model, although not statistically significant and thus not presented in the table.

^b ^Log likelihood.

^c ^Second-order penalized quasi-likelihood (PQL) estimates with restricted iterative generalised square algorithm.

^d ^Markov-chain Monte Carlo (MCMC) estimates.

**Table 5:** Odds ratios (ORs) with 95% confidence intervals (CIs) for the explanatory variables^a ^significantly associated with veterinary treatment (yes = 1/no = 0), given a disease event (two-level logistic model) for young animals estimated using log likelihood estimation.

Variables	Categories	OR	95% CI	
Age	< 2	BL^b^		
	2 to 15	1.9	0.6	5.8
	> 15	7.5	1.9	30.1
Another animal with an event at the same date	Yes	3.7	1.4	9.5
	No	BL		
Disease complex	Lameness	11.4	2.3	56.0
	Other	6.1	1.8	20.8
	Cough	4.4	1.1	18.0
	Gastro-intestinal	BL		
Good record-keeping ability	Yes	BL		
	No	10.4	1.6	66.9

^a ^Study month was identified as a confounder and was therefore also included in the model, although not statistically significant and thus not presented in the table.

^b ^Baseline

Moreover, the random herd effect varied between the LL, the PQL and the MCMC procedures (Table 2) and was significant in the LL and MCMC procedures, but not in the PQL procedure. The estimated variation ranged from 30–46%.

## Discussion

### Fixed part

This study deals with the probability of receiving veterinary treatment (i.e. the probability that the farmer contacted the veterinarian) for diseased animals. Thus, it is important to keep in mind that the explanatory variables significantly associated with the probability of receiving veterinary treatment are variables that seems to influence whether diseased animals receives veterinary treatment or not. Hence, they are not necessarily risk factors for disease.

### Cows

Breed was the only statistically significant herd-level characteristic that affected the cow's probability of receiving veterinary treatment, with lower odds in SR breeds than in SH breeds. Several studies based on either farmer's disease records or veterinary records have found a difference in incidence of disease between breeds [16-20]. This difference could have many possible explanations. The concentration of several blood variables have been found to differ around calving in primiparous cows of SH and SR breed and potentially explain why cows of SH-breed have higher disease incidence [21]. It could also be hypothesised that a difference in immune response between breeds could affect the severity of a disease event and thus the odds of receiving veterinary treatment. Nyman et al. [1] found that herds with high incidence rates of clinical mastitis consisted more often of SH cows, and in these herds, the farmer more often contacted a veterinarian as soon as the cow's milk appearance was altered than in herds with low incidence rates. Persson Waller and her colleagues [19] suggested that differences in treatment strategies between SR and SH herds could have biased the effect of breed in studies using veterinary records of disease, concurring with our findings.

The higher odds for veterinary treatment early in lactation could be expected, because the transition period (from three weeks before until three weeks after calving) is known to be associated with a higher risk of disease [22,23]; and especially as many diseases during this period have an acute course and demand veterinary assistance or treatment. Metabolic and physical stress related to pregnancy, parturition and lactation have been described to have a negative impact on the health [24]. It is also possible that changes in the immune system during this time cause a more severe course of disease. Further, it is also possible that the lower odds for veterinary treatment later in lactation was affected by different treatment strategies for cows in different lactational stages, as has been shown for mastitis by Vaarst and her colleagues [2].

The interaction between diagnosis and whether or not there was another animal with an event at the same date resulted in the finding that there were higher odds for treatment of lameness, metabolic disorders, udder disorders and other disorders if there was another animal with an event at the same date. Animals with an event at the same date as another animal belonged to herds with a significantly higher herd size (p < 0.001, using the Wilcoxon rank-sum test, data not shown). It is likely that the veterinarian was consulted for milder disease events to a higher degree if he or she was contacted for another event. The cost for examining, and treating, a mild case will be lower when the veterinarian is already at the farm. A higher incidence of veterinary treatments in large herds could therefore be an effect of a higher number of events near in time in large herds, and not only an effect of a higher incidence of disease in larger herds. On the other hand, cows in smaller herd have a larger relative economic value and could therefore be more likely to receive veterinary treatment than cows in larger herds, as discussed by Østerås et al. [25]. A difference in a variable at the individual level, i.e. the probability of veterinary treatment in the case of a disease event that relates to the group to which the individual belongs, is called a contextual effect [26]. Contextual effects that are not accounted for could lead to false inferences, as shown by Stryhn and his colleagues [27]. Herd size as well as herd main breed should therefore be regarded, and taken into account, as contextual effects.

Significant differences in odds for veterinary treatment between disease complexes were mainly found between lameness and the other disease complexes, with lameness having lower odds for veterinary treatment. For most events of lameness that were not veterinary treated, the farmer had reported that the hoof trimmer had been contacted. There is a voluntary hoof health register in Sweden, and a combination of the disease database and the hoof health register would give more complete information on hoof disorders. Reproductive disorders had significantly lower odds for veterinary treatment than udder disorders and other disorders in presence of another animal with a disease event at the same day. This could be seen as an indication of that those events of udder disorders and other disorders were milder and more likely to be consulted for only when the veterinarian was already at the farm.

Study month had an effect on the odds for veterinary treatment. Whether the animals are kept on pasture or housed indoors could affect the farmers' treatment strategy, as well as the ability to detect disease. Although our results could not reveal a seasonal pattern, a seasonal variation in the severity of the disease events and a seasonal difference in pathogen prevalence have been reported, for example for mastitis [28-30]. A higher incidence of parturient paresis has also been found during grazing [31]. It is, however, also possible that the differences between study months are an effect of our study design. January, the month when there is least to do at a farm under Swedish conditions, was the first study month, while October was the last. A lack of time during the harvest and a reduced interest in the study during the last months could have influenced the farmers' own recordings in favour of those events that resulted in veterinary treatment. Such events may be easier to recall, because the veterinarian leaves documentation on the farm after the treatment.

### Young animals

Our study found higher odds for veterinary treatment for lameness and other disorders compared to diarrhoea. In a recent study by Svensson et al. [32], 68% of the cases of diarrhoea and 46% of the cases of respiratory disease were mild. A low probability of receiving veterinary treatment could therefore be expected because of a high proportion of events of diarrhoea with mild clinical signs. The difference between age categories is most likely explained by the different diseases affecting young calves and older animals. For example, of the 149 disease events in animals of an age < 2 months, 81 (54%) were events of diarrhoea and 41 (28%) were events of coughing. As some diseases are more likely to affect animals in a specific age group, the variable disease complex could be seen as appearing between age and the farmers decision of veterinary treatment on the causal pathway, i.e. it is a so called intervening variable. By keeping such a variable in the model the estimates of age are at risk of being incorrect. When estimating the model without the disease complex, the odds for veterinary treatment in the LL procedure for animals at the age of 2–15 months and above 15 months were higher than for animals of an age < 2 months (OR 3.1; 95% CI 1.1, 9.0 and OR 24; 95% CI 7.2, 77, respectively). Events treated on the same date as another animal with an event increased the odds for veterinary treatment. The reason for this is the same as that for the corresponding finding in the cow model.

### Consequences for studies based on veterinary reported data

This study has identified a number of factors that affects whether a diseased animal receives veterinary treatment or not. From the data it was not possible to fully distinguish severe disease event from those with milder symptoms of disease but it is likely that many of those not veterinary treated were milder disease events. It is also likely that for some events that were not veterinary treated the farmers decided to slaughter the animal instead of treating it. When using veterinary treated disease event in studies of risk factors for disease, the estimates of breed, lactational stage and likely also estimates of herd size could be biased. A number of variables were considered for inclusion in the models, but were not significantly associated with veterinary treatment such as housing types and milk yield (cow or herd average). Hence, based on our results, veterinary recording data could be used to study those risk factors without the risk of bias being introduced because of a differential veterinary treatment attributed to the risk factors being investigated.

### Random part

The original data consisted of events subclustered within individuals which in turn were clustered within herd. Because only 16% of the cows had more than one event, multiple events in animals were removed. The clustering of events within herds was accounted for by the random effect of herd which was significant in the cow model, with similar results from the different estimation procedures. In the model for young animals, the estimation procedures showed different results, and the random effect of herd was only significant for the LL and MCMC procedures. It is also possible that the random effect was over-estimated due to some herds having only events that resulted in veterinary treatment. Excluding these herds from the model reduced the herd-level variation to between 25% and 28% for cows and 24% and 39% for young animals (data not shown). It was, however, not possible to determine if the farmers whose herds only had events that had resulted in veterinary treatment had reported all the events or had failed to report events that had not resulted in veterinary treatment.

In the cow analysis, 39–42% of the variation was at the herd level. In the present study we had no information about the farmer; rather, their influence can be considered to be part of the herd effect. Thus our results are in line with the results in Vaarst [2], who found that the choice of veterinary treatment was influenced by the farmer, as they put different weight on a number of cow characteristics. A recent study has evaluated the extent to which mastitis incidence could be explained by farmers' behaviour and attitude [33]. It was found that self-reported behaviour and attitudes combined explained 29% of the variation in clinical mastitis between herds. Further, the culling strategy, the number of person-years devoted to dairy herd management, and the treatment strategy after the observation of single clots have been found to influence the incidence of clinical mastitis [34]. Differences in the thresholds for treatment and the choice of diagnoses have also been found in interviews with practising veterinarians who treat cattle, indicating that veterinarians are another source of variation [35].

Our results show that a substantial part of the variation in the odds of veterinary treatment concerns variation between herds. Hence, future studies are also needed to explore effects of the characteristics and attitudes of farmers and veterinarians, as well as the expected economic value of different treatment strategies.

### Preventing bias

In the present study, only one (randomly chosen) diagnostic event per animal was included and this event was only taken from herds with at least four diagnostic events in either cows or young animals. The reason for this is that small groups (few events per herd in our study) have been shown to result in biased estimates in multi-level models [36,37]. For example, when evaluating bias in clustered data, Clarke [36] found that two-level models with a group average of five produced unbiased estimates both for fixed and random effects. With a group average ≤ 2, the two-level model produced a downward bias in fixed effects and an upward bias in the random effect.

In the present study, the models were estimated using three different estimation procedures: the LL, the PQL and the MCMC procedures. The estimates from the different procedures were similar for the cow model and for the fixed effects in the young animal model. Multilevel models with binary outcomes have been shown to produce biased estimates [37] and the different estimation procedures used has different advantages, and disadvantages. While it has been found that the MCMC procedure produces less biased results, the LL procedure is preferable during model building since the contribution of the potential explanatory variables can be evaluated using the likelihood ratio test. Further, the PQL procedure is preferable for the evaluation of the model fit. Because the methods available in multilevel modelling, to the authors' knowledge, have different benefits and limitations, several estimation procedures could be used to ensure confidence in the results in multilevel modelling. This is an approach that has been adopted previously [38,39].

In studies based on disease events reported by veterinarians or cattle owners, a misclassification (that animals are classified as healthy when there are no records saying they are diseased) bias is likely to be present [11,40]. In our previous work [12] the proportion of events missing in the farmers' data, but reported to the DDD by veterinarians, was 0.12. We found it likely that those farmers who failed to report events to us that had resulted in veterinary treatment, also, to a greater extent, failed to report events that had not resulted in veterinary treatment. We therefore defined criteria for what we thought was an acceptable loss of data and included the variable 'good record-keeping ability' in the analysis. This variable was significantly associated with the OR for veterinary treatment in the young animal model, but not in the cow model.

As discussed previously, events resulting in veterinary treatment may be easier to recall because the farmers have a copy of the veterinary record. It is therefore more likely that the farmers forgot to report events where the animal did not receive veterinary treatment than events that did result in veterinary treatment. This could have affected our results and more studies are needed to validate our results and assess the extent of this recall bias.

The farmers gave, in general, a more detailed description of the disease event if a veterinarian was not contacted, indicating that the farmers were relying on the veterinarians' diagnosis in cases where the veterinarians was contacted. The thorough description of disease events not receiving veterinary treatment enabled the further categorisation of disease events diagnosed as "other disease" by the farmer.

## Conclusion

Our results show that whether or not an animal receives veterinary treatment in the case of a disease event depends on both cow and herd characteristics, such as the lactational stage, the animal's age and the herd's breed and whether there was another disease event in the herd at the same day. The sources of differential misclassification of disease events, which was identified in the present study, could bias results and inferences in studies on disease events resulting in veterinary treatment.

## Competing interests

The authors declare that they have no competing interests.

## Authors' contributions

MJM designed the study in cooperation with authors AL, IV and AE. MJM further performed the statistical analyses, interpreted the data and drafted the manuscript. AL, UE and AE participated in the statistical analyses and revised the manuscript. IV revised the manuscript. All authors have read and approved the final manuscript.

## References

[B1] Nyman A-K, Ekman T, Emanuelson U, Gustafsson AH, Holtenius K, Persson Waller K, Hallén Sandgren C (2007). Risk factors associated with the incidence of veterinary-treated clinical mastitis in Swedish dairy herds with a high mik yield and a low prevalence of subclinical mastitis. Preventive Veterinary Medicine.

[B2] Vaarst M, Paarup-Laursen B, Houe H, Fossing C, Andersen HJ (2002). Farmers' choice of medical treatment of mastitis in Danish dairy herds based on qualitative research interviews. Journal of Dairy Science.

[B3] Groenendaal H, Galligan DT, Mulder HA (2004). An Economic Spreadsheet Model to Determine Optimal Breeding and Replacement Decisions for Dairy Cattle. J Dairy Sci.

[B4] Ortman K, Svensson C (2004). Use of antimicrobial drugs in Swedish dairy calves and replacement heifers. Vet Rec.

[B5] Mulder CAT, Bonnett BN, Martin SW, Lissemore K, Page PD (1994). The Usefulness of the Computerized Medical Records of One Practice for Research into Pregnancy Loss in Dairy-Cows. Prev Vet Med.

[B6] Carlen E, Schneider MdP, Strandberg E (2005). Comparison Between Linear Models and Survival Analysis for Genetic Evaluation of Clinical Mastitis in Dairy Cattle. J Dairy Sci.

[B7] Egenvall A, Bonnett B, Wattle O, Emanuelson U (2008). Veterinary-care events and costs over a 5-year follow-up period for warmblooded riding horses with or without previously recorded locomotor problems in Sweden. Prev Vet Med.

[B8] Maizon DO, Oltenacu PA, Gröhn YT, Strawderman RL, Emanuelson U (2004). Effects of diseases on reproductive performance in Swedish Red and White dairy cattle. Prev Vet Med.

[B9] Iezzoni LI (1997). Assessing quality using administrative data. Ann Intern Med.

[B10] Lawrenson R, Williams T, Farmer R (1999). Clinical information for research; the use of general practice databases. J Public Health Med.

[B11] Olsson SO, Baekbo P, Hansson SO, Rautala H, Osteras O (2001). Disease recording systems and herd health schemes for production diseases. Acta Vet Scand.

[B12] Mörk M, Lindberg A, Alenius S, Vågsholm I, Egenvall A (2009). Comparison between dairy cow disease incidence in data registered by farmers and in data from a disease-recording system based on veterinary reporting. Preventive Veterinary Medicine.

[B13] Brolund L (1990). Technical Utilization of Cell Count in the Milk-Recording Service (Cellhaltens tekniska utnyttjande i kokontrollen). Meddelande 161, 40–41.

[B14] Dohoo I, Martin SW, Stryhn H (2003). Veterinary Epidemologic Research.

[B15] Rasbash J, Steele F, Browne WJ, Goldstein H (2009). A User's Guide to MLwiN, v210.

[B16] Bendixen PH, Vilson B, Ekesbo I, Astrand DB (1987). Disease Frequencies in Dairy-Cows in Sweden .2. Retained Placenta. Prev Vet Med.

[B17] Bendixen PH, Vilson B, Ekesbo I, Astrand DB (1988). Disease Frequencies in Dairy-Cows in Sweden .5. Mastitis. Prev Vet Med.

[B18] Bendixen PH, Vilson B, Ekesbo I, Åstrand DB (1988). Disease frequencies in dairy cows in Sweden. VI. Tramped teat. Prev Vet Med.

[B19] Persson Waller K, Bengtsson B, Lindberg A, Nyman A, Ericsson Unnerstad H (2009). Incidence of mastitis and bacterial findings at clinical mastitis in Swedish primiparous cows – Influence of breed and stage of lactation. Vet Microbiol.

[B20] Schnier C, Hielm S, Saloniemi HS (2004). Comparison of the disease incidences of Finnish Ayrshire and Finnish Black and White dairy cows. Prev Vet Med.

[B21] Nyman AK, Emanuelson U, Holtenius K, Ingvartsen KL, Larsen T, Waller KP (2008). Metabolites and immune variables associated with somatic cell counts of primiparous dairy cows. Journal of Dairy Science.

[B22] Drackley JK (1999). Biology of Dairy Cows During the Transition Period: the Final Frontier. J Dairy Sci.

[B23] Ingvartsen KL, Dewhurst RJ, Friggens NC (2003). On the relationship between lactational performance and health: is it yield or metabolic imbalance that cause production diseases in dairy cattle? A position paper. Livest Prod Sci.

[B24] Mallard BA, Dekkers JC, Ireland MJ, Leslie KE, Sharif S, Vankampen CL, Wagter L, Wilkie BN (1998). Alteration in immune responsiveness during the peripartum period and its ramification on dairy cow and calf health. Journal of Dairy Science.

[B25] Østerås O, Solbu H, Refsdal AO, Roalkvam T, Filseth O, Minsaas A (2007). Results and evaluation of thirty years of health recordings in the Norwegian dairy cattle population. Journal of Dairy Science.

[B26] Snijders TAB, Bosker RJ (1999). Multilevel Analysis: An Introduction to Basic and Advanced Multilevel Modeling.

[B27] Stryhn H, De Vliegher S, Barkema HW (2006). Contextual multilevel models: Effects and correlations at multiple levels. Proceedings of the 11th International Symposium on Veterinary Epidemiology and Economics Cairns, Australia.

[B28] Dohoo IR, Martin SW, Mcmillan I, Kennedy BW (1984). Disease, Production and Culling in Holstein-Friesian Cows .2. Age, Season and Sire Effects. Prev Vet Med.

[B29] Hogan JS, Smith KL, Hoblet KH, Schoenberger PS, Todhunter DA, Hueston WD, Pritchard DE, Bowman GL, Heider LE, Brockett BL, Conrad HR (1989). Field Survey of Clinical Mastitis in Low Somatic-Cell Count Herds. Journal of Dairy Science.

[B30] Riekerink RGMO, Barkema HW, Stryhn H (2007). The effect of season on somatic cell count and the incidence of clinical mastitis. J Dairy Sci.

[B31] Bendixen PH, Vilson B, Ekesbo I, Åstrand DB (1987). Disease frequencies in dairy cows in Sweden. III. Parturient paresis. Prev Vet Med.

[B32] Svensson C, Lundborg K, Emanuelson U, Olsson SO (2003). Morbidity in Swedish dairy calves from birth to 90 days of age and individual calf-level risk factors for infectious diseases. Prev Vet Med.

[B33] Jansen J, Borne BHPvd, Renes RJ, van Schaik G, Lam TJGM, Leeuwis C (2008). Mastitis incidence explained by farmers' attitude and behaviour. Society for Veterinary Epidemiology and Preventive Medicine Proceedings of a meeting held at Liverpool, UK, on the 26th–28th March 2008.

[B34] Barnouin J, Bord S, Bazin S, Chassagne M (2005). Dairy management practices associated with incidence rate of clinical mastitis in low somatic cell score herds in France. J Dairy Sci.

[B35] Kristensen E, Nielsen DB, Jensen LN, Vaarst M, Enevoldsen C (2008). A mixed methods inquiry into the validity of data. Acta Vet Scand.

[B36] Clarke P (2008). When can group level clustering be ignored? Multilevel models versus single-level models with sparse data. J Epidemiol Community Health.

[B37] Goldstein H, Rasbash J (1996). Improved approximations for multilevel models with binary responses. Journal of the Royal Statistical Society Series A – Statistics in Society.

[B38] Dohoo IR, Tillard E, Stryhn H, Faye B (2001). The use of multilevel models to evaluate sources of variation in reproductive performance in dairy cattle in Reunion Island. Preventive Veterinary Medicine.

[B39] Vigre H, Dohoo IR, Stryhn H, Busch ME (2004). Intra-unit correlations in seroconversion to Actinobacillus pleuropneumoniae and Mycoplasma hyopneumoniae at different levels in Danish multi-site pig production facilities. Preventive Veterinary Medicine.

[B40] Kadarmideen HN (2002). Recording disease and reproductive events in dairy cattle for developing national health and fertility selection indices. Performance recording of animals: state of the art 2002 Proceedings of the 33rd Biennial Session of ICAR Interlaken, Switzerland.

